# Population Genomic Structure and Genome-Wide Linkage Disequilibrium in Farmed Atlantic Salmon (*Salmo salar* L.) Using Dense SNP Genotypes

**DOI:** 10.3389/fgene.2018.00649

**Published:** 2018-12-14

**Authors:** Agustin Barria, Maria E. López, Grazyella Yoshida, Roberto Carvalheiro, Jean P. Lhorente, José M. Yáñez

**Affiliations:** ^1^Facultad de Ciencias Veterinarias y Pecuarias, Universidad de Chile, La Pintana, Chile; ^2^Faculdade de Ciências Agrárias e Veterinárias, Universidade Estadual Paulista Júlio de Mesquita Filho, Jaboticabal, Brazil; ^3^Benchmark Genetic S.A., Puerto Montt, Chile; ^4^Nucleo Milenio INVASAL, Concepción, Chile

**Keywords:** linkage disequilibrium, *Salmo salar*, selective breeding, GWAS, population structure

## Abstract

Chilean Farmed Atlantic salmon (*Salmo salar*) populations were established with individuals of both European and North American origins. These populations are expected to be highly genetically differentiated due to evolutionary history and poor gene flow between ancestral populations from different continents. The extent and decay of linkage disequilibrium (LD) among single nucleotide polymorphism (SNP) impacts the implementation of genome-wide association studies and genomic selection and provides relevant information about demographic processes of fish populations. We assessed the population structure and characterized the extent and decay of LD in three Chilean commercial populations of Atlantic salmon with North American (NAM), Scottish (SCO), and Norwegian (NOR) origin. A total of 123 animals were genotyped using a 159 K SNP Axiom^®^ myDesign^TM^ Genotyping Array. A total of 32 K SNP markers, representing the common SNPs along the three populations after quality control were used. The principal component analysis explained 78.9% of the genetic diversity between populations, clearly discriminating between populations of North American and European origin, and also between European populations. NAM had the lowest effective population size, followed by SCO and NOR. Large differences in the LD decay were observed between populations of North American and European origin. An *r*^2^ threshold of 0.2 was estimated for marker pairs separated by 7,800, 64, and 50 kb in the NAM, SCO, and NOR populations, respectively. In this study we show that this SNP panel can be used to detect association between markers and traits of interests and also to capture high-resolution information for genome-enabled predictions. Also, we suggest the feasibility to achieve similar prediction accuracies using a smaller SNP data set for the NAM population, compared with samples with European origin which would need a higher density SNP array.

## Background

Atlantic salmon (*Salmo salar*) is one of the species of farmed fish with the highest commercial value in aquaculture (FAO, 2016a). Chile is the second largest producer, generating nearly 532,000 tons of this specie in 2016 (FAO, 2016b). All of the Atlantic salmon populations farmed in Chile were introduced from three main geographical origins (i) North America, (ii) Scotland, and (iii) Norway. These populations also represent the main origins of cultured Atlantic salmon worldwide. Breeding programs for Atlantic salmon were first established in Norway during the early 1970s (Gjedrem et al., 2012). Since then, there has been an increased interest in implementing genetic improvement programs for salmon in the most important producer countries, including Australia, Chile, Iceland, Ireland, Scotland and Norway. The main traits included in the breeding objectives of Atlantic salmon are growth, disease resistance, carcass quality and age at sexual maturation (Rye et al., 2010).

Recent advances in next-generation sequencing and high-throughput genotyping technologies have allowed the development of valuable genomic resources in aquaculture species (Yáñez et al., 2015). For instance, dense single nucleotide polymorphism (SNP) panels have been developed for Atlantic salmon (Houston et al., 2014; Yáñez et al., 2016). Genetic evaluations for traits that are difficult to measure in selection candidates, such as disease resistance and carcass quality traits, can be more accurate when integrating genome-wide SNP information, in what has been called genomic selection (Meuwissen et al., 2001; Sonesson and Meuwissen, 2009). Genomic selection exploits the linkage disequilibrium (LD) that exists between SNP and quantitative trait loci (QTL) or causative mutations that are involved in the variation of the trait (Goddard and Hayes, 2009), increasing the accuracy of genome-enabled estimated breeding values (GEBVs) in farmed salmon species (Ødegård et al., 2014; Tsai et al., 2016; Bangera et al., 2017; Vallejo et al., 2017; Yoshida et al., 2018; Barria et al., 2018b). Furthermore, association mapping through genome wide association studies (GWAs) is a useful approach to detect genomic regions and genes involved in economically important traits for salmon aquaculture and they also rely on LD between the QTL and SNP markers. Thus an adequate SNP density is required to assure that all QTL are in LD with a marker (Flint-Garcia et al., 2003).

In addition, knowing the extent and pattern of LD can be used to help explore different evolutionary forces that may affect certain regions of the genome (Ardlie et al., 2002). Because it is affected by population growth, genetic drift, admixture or migration, population structure, variable recombination rates and artificial/natural selection, LD can be variable among populations and loci (Ardlie et al., 2002). Different measures of LD between two loci have been proposed, among them the absolute value of *D’* (also called Lewontin’s *D*’) and *r*^2^ are the most widely used. *D’* = 1, indicates no recombination between loci and complete LD, while values less than 1 indicate that loci have been separated by recombination. *D*’ estimations are overestimated in small sample sizes and low frequencies of minor allele, therefore, high values of *D’* can be obtained even when markers are in linkage equilibrium (Ardlie et al., 2002). Therefore, *r*^2^, the squared correlation between alleles at two loci, is the most accepted measure for comparing and quantifying LD (Pritchard and Przeworski, 2001).

To date, several studies have been performed to determine the levels and extent of LD in livestock species such as dairy (Khatkar et al., 2008; Bohmanova et al., 2010), beef cattle (McKay et al., 2007; Lu et al., 2012; Espigolan et al., 2013; Porto-Neto et al., 2014), pigs (Badke et al., 2012; Ai et al., 2013), goats (Mdladla et al., 2016; Visser et al., 2016), and sheep (Prieur et al., 2017). Moreover, some studies have related patterns of LD with genomic regions subjected to selection in domestic species (Prasad et al., 2008). Recent studies have also aimed at characterizing the levels of LD in farmed aquaculture species, such as rainbow trout (Rexroad and Vallejo, 2009; Vallejo et al., 2018), coho salmon (Barria et al., 2018a), and Atlantic salmon (Kijas et al., 2017). However, until now there have been no comprehensive studies aiming at characterizing and comparing levels and extent of LD in commercial Atlantic salmon populations that include the three main geographical origins. The goal of this study was to (a) assess the levels of LD in farmed Atlantic salmon populations with three different geographical origins (i.e., Canada, Scotland, and Norway); (b) calculate the effective population size for each breeding population; and (c) estimate the population structure and genetic admixture of each population.

## Materials and Methods

### Populations and Samples

The current study is comprised of 123 Atlantic salmon individuals from three different commercial populations cultivated in the South of Chile, which have different geographical origins. These fish were obtained from Chilean farmed populations, which were originated from imported stocks. The Norwegian population was comprised of 43 fish belonging to a breeding population derived from the Mowi strain, which is the oldest farmed population constituted in Norway. This strain was established in the late 1960s using fish from west coast rivers in Norway (NOR), River Bolstad in the Vosso watercourse, River Årøy and Maurangerfjord area (Verspoor et al., 2007). Ova of this strain were introduced into Ireland from 1982 to 1986 (Norris et al., 1999) and from there, they were imported to Chile for farming purposes in the 1990s (Solar, 2009). Since 1997 this population has been selected for rapid growth in Chile (Yáñez et al., 2013, 2014; Correa et al., 2015, 2017). A second population of 43 fish of Scottish origin (SCO) was comprised of samples from a strain derived from fish from Loch Lochy, located on the West Coast of Scotland. Fish of this strain are described as a stock with rapid growth potential and a high early maturation grilsing rate (Johnston et al., 2000). During the 1980s, eggs from the Scottish population were introduced to Chile to establish an aquaculture broodstock. The third population used in this study was comprised of 37 fish of North American (NAM) origin; belonging to a domestic strain established in the 1950s, using ova from the Gaspé Bay (QC, Canada). It is presumed that fish of this strain were transferred and kept at an aquaculture hatchery located in the state of Washington, United States for two generations. Fertilized eggs of this strain were introduced from Washington to Chile between 1996 and 1998 (López et al., 2018). Since their introduction in Chile, these lines have been maintained separately and no crosses between them have been performed. The mean relatedness among individuals was estimated within each population using Plink v1.90 (Purcell et al., 2007). The estimated values were 0.18 (0.19), 0.05 (0.08), and 0.05 (0.06) for the NAM, SCO, and NOR populations, respectively.

### Genotyping

Fin clip samples from individuals from the three populations were obtained for genomic DNA extraction and further genotyping. Genotyping was carried out using a 200 K Affymetrix Axiom^®^ myDesign Custom Array as described by Yáñez et al. (2016). This dense SNP array contains 151,509 polymorphic SNPs with unique position and evenly distributed markers across the genome. A total of 2,302 (1.6%) SNPs were discarded prior to analysis due to unknown chromosomal location on the *S. salar* reference genome (Yáñez et al., 2016). Quality control of genotypes was performed using Axiom Genotyping Console (AGT, Affymetrix) and SNPolisher for R, according to the Best Practices procedures indicated by the array manufacturer^1^. Quality control (QC) was performed using PLINK software v1.09, and assessed separately for each population. SNPs with minor allele frequency (MAF) lower than 5%, significantly deviating from Hardy–Weinberg Equilibrium (HWE) (*p* < 1e-6), and a SNP call rate of lower than 95% were excluded. Samples with more than 5% of missing genotypes were also excluded. All subsequent analyses were done using the common SNPs along the three populations after QC.

### Population Structure and Genetic Admixture Analysis

To investigate genetic structure among populations, we performed a principal component analysis (PCA) implemented in PLINK v1.09. Visualization of the first two PCA were plotted along two axes in R. Additionally, we used a hierarchical Bayesian modeling implemented in STRUCTURE software, using a burn-in of 20,000 iterations, and running 50,000 iterations with three replicates. Subsequently, we computed the posterior probability of each K value according Pritchard et al. (2000), to choose the best K assuming a uniform prior on K between 1 and 10.

### Estimation of LD

Linkage disequilibrium as Pearson’s squared correlation coefficient (*r*^2^) was chosen over |*D’*| to predict the LD between each pair of molecular markers. This statistic is less sensitive to bias caused by differences in allelic frequencies (Ardlie et al., 2002), more appropriate for biallelic markers (Zhao et al., 2005) and can be used to compare the results with previous studies in salmonid species and other domestic animals. Genotypes were coded as 2, 1, and 0 in function of the number of non-reference alleles. The pair-wise LD as *r*^2^ was calculated for each population and within chromosomes using Plink v1.09 using the formula proposed by Hill and Robertson (Hill and Robertson, 1968). For each SNP pair, bins of 100 kb were created based on pairwise physical distance. The extent and decay of the LD, was visualized by plotting the average *r*^2^ within each bin from 0 up to 10 Mb, using R software (R Core Team, 2016).

### Effective Population Size

Historical effective population size (*N*_e_) was estimated using SNeP v1.1 (Barbato et al., 2015). SNeP software estimates *N*_e_ using LD data calculated through the following formula proposed by Corbin et al. (2012):

$$N_{\text{t}}\;=\frac{1}{\left(4f\left(c_{t}\right)\;\right)}\;\;(\frac{1}{E\left[r_{\text{adj}}^{2}|\;c_{t}\right]}\;-\;\alpha )$$

Where N_t_ is the effective population size t generations ago, c_t_ is the recombination rate, t generations ago, being proportional to the physical distance between SNP markers, *r*^2^_adj_ is the estimated LD adjusted for sample size and α is the adjustment for mutation rate. As proposed by Tenesa et al. (2007), we used an α = 2, considering that mutation does occurs. The minimum and maximum distance used between SNPs for *N*_e_ estimation was 0 and 5 Mb, respectively. Data was grouped in 30 distance bins of 50 kb each. Finally, *N*_e_ was estimated from the *r*^2^ values calculated for the mean distance of each distance bin. Considering the relative small number of SNPs per chromosome, the estimated *N*_e_ per chromosome was calculated using harmonic mean (Alvarenga et al., 2018). Contemporary effective population size for each population, was estimated using NeEstimator v2.01 (Do et al., 2014). Briefly, estimation was based on LD method, with a critical value (Pcrit) of 0.05 and a non-random mating model.

## Results

### SNP Quality Control

No animals from the three populations were removed after quality control, giving genotype data from 123 individuals (37, 43, and 43 from NAM, SCO, and NOR, respectively). A total of 40,316 (27.02%), 113,282 (75.92%), and 136,446 (91.46%) SNP markers passed the QC criteria for the NAM, SCO and NOR populations, respectively. Filtered SNPs differed significantly between populations of North American or European origin. 106 K SNPs were excluded from the NAM population by a low MAF, representing 70% of the total available markers in the array. The markers excluded by MAF in SCO and NOR populations reached 23 and 7.8%, respectively. A summary of the number of fish genotyped from each population, number of SNPs excluded by HWE, MAF and the final number of SNPs per population are shown in Table 1. After QC, a total of 31,978 common SNPs among the three populations were identified. These 32 K SNPs were used for all the subsequent analyzes.

**Table 1:** Summary of results from quality control of SNPs for each farmed population genotyped with the 200K SNP array.

Population origin^1^	n_g_^2^	Call rate^3^	HWE^4^	MAF < 0.05^5^	Final number of SNPs^6^
					
NAM	37	2600	188	106103	40316
SCO	43	923	62	34940	113282
NOR	43	994	87	11660	136466


*^*1*^Geographical origin of each farmed population: NAM, North America; SCO, Scotland; NOR, Norway.*

*^*2*^Number of genotyped samples.*

*^*3*^Number of excluded SNPs by the genotype call rate <0.95.*

*^*4*^Number of excluded SNPs by Hardy–Weinberg Equilibrium.*

*^*5*^Number of excluded SNPs with minor allele frequency <0.05.*

*^*6*^Number of SNPs retained for each population.*

### Summary Statistics for Each Population

Summary statistics of each chromosome’s length, average *r*^2^ and *N*_e_ estimates among SNPs for each chromosome and population are shown in Table 2. The markers spanned 2,218.6 Mb, of the Atlantic salmon genome, encompassing 70% of the total sequence length (assuming a *S. salar* genome size of 2.96 Gb based on the last assembly GCA_000233375.4). Average *r*^2^ between adjacent SNPs reached up to 0.26 ± 0.28 in the NAM population. These values were higher than for SCO and NOR populations (0.11 ± 0.14 and 0.07 ± 0.10, respectively). The average LD, measured as *r*^2^, between adjacent markers across the 29 chromosomes, ranged from 0.16 to 0.35, 0.08 to 0.14, and 0.05 to 0.09 in NAM, SCO, and NOR populations, respectively (Table 2). These results indicate that average levels of LD among syntenic SNPs are considerably lower in both populations with European origin compared with the population with North American origin. Also, LD for the SCO population is slightly higher when compared with the NOR population. For each population, effective population size by chromosome was calculated up to 180 generations ago, excepting for *Ssa08*, *Ssa26*, and *Ssas28* in which *N*_e_ was estimated up to 55 generations ago. Estimations were lower for all chromosomes in NAM population, while the higher values were estimated in the population with Norwegian origin (Table 2).

**Table 2:** Estimated chromosome length and average linkage disequilibrium values for three Chilean farmed populations of Atlantic salmon with Norwegian (NOR), Scottish (SCO), and North American (NAM) origin.

Chr^a^		NAM		SCO		NOR	
Ssa	Length (Mb)	Average *r*^2^	*N_e_*^b^	Average *r*^2^	*N_e_*	Average *r*^2^	*N_e_*
1	158.62	0.32 (0.32)	22	0.10 (0.13)	106	0.08 (0.12)	135
2	72.29	0.16 (0.21)	51	0.08 (0.12)	130	0.07 (0.10)	165
3	91.90	0.31 (0.33)	23	0.08 (0.13)	128	0.06 (0.10)	218
4	80.74	0.22 (0.27)	34	0.14 (0.17)	65	0.07 (0.11)	170
5	77.05	0.20 (0.24)	37	0.09 (0.12)	118	0.06 (0.09)	206
6	86.50	0.21 (0.26)	40	0.08 (0.12)	131	0.07 (0.11)	171
7	56.13	0.20 (0.23)	40	0.08 (0.11)	141	0.05 (0.08)	237
8	23.97	0.35 (0.35)	23	0.08 (0.12)	113	0.08 (0.11)	120
9	141.51	0.34 (0.31)	16	0.12 (0.15)	91	0.07 (0.11)	191
10	116.05	0.23 (0.27)	39	0.11 (0.14)	94	0.09 (0.12)	141
11	93.49	0.26 (0.28)	28	0.11 (0.15)	90	0.09 (0.13)	138
12	91.01	0.21 (0.24)	44	0.13 (0.16)	71	0.07 (0.10)	177
13	107.38	0.30 (0.30)	26	0.12 (0.17)	86	0.09 (0.13)	136
14	93.46	0.23 (0.28)	45	0.11 (0.15)	101	0.09 (0.13)	129
15	102.94	0.31 (0.31)	26	0.12 (0.16)	87	0.08 (0.12)	147
16	86.10	0.25 (0.28)	37	0.13 (0.16)	83	0.09 (0.13)	134
17	55.49	0.24 (0.27)	29	0.11 (0.14)	103	0.06 (0.10)	214
18	69.96	0.30 (0.31)	29	0.16 (0.20)	56	0.07 (0.12)	173
19	82.01	0.20 (0.24)	39	0.10 (0.14)	106	0.09 (0.14)	122
20	86.32	0.31 (0.31)	18	0.13 (0.17)	77	0.08 (0.12)	158
21	57.47	0.26 (0.29)	30	0.08 (0.12)	140	0.06 (0.10)	227
22	62.97	0.21 (0.26)	42	0.09 (0.13)	119	0.07 (0.11)	159
23	49.59	0.36 (0.33)	16	0.10 (0.13)	108	0.05 (0.08)	257
24	47.63	0.30 (0.30)	19	0.11 (0.15)	80	0.06 (0.10)	197
25	51.27	0.26 (0.28)	30	0.13 (0.17)	63	0.08 (0.12)	148
26	47.45	0.22 (0.25)	30	0.11 (0.15)	66	0.07 (0.11)	124
27	43.45	0.21 (0.27)	38	0.10 (0.13)	117	0.05 (0.08)	247
28	38.60	0.23 (0.26)	27	0.09 (0.13)	90	0.07 (0.10)	141
29	42.38	0.17 (0.23)	53	0.09 (0.13)	97	0.06 (0.09)	188


*SD in parenthesis. ^*a*^Chromosome; ^*b*^effective population size.*

**FIGURE 1:**
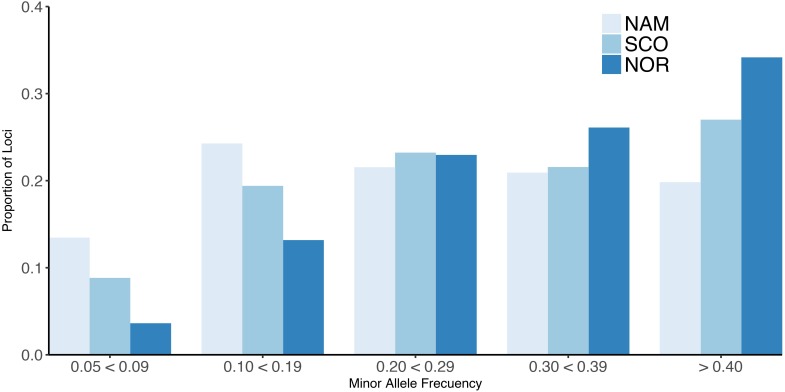
Minor allele frequency (MAF) for farmed Atlantic salmon populations. Proportion of loci for different values of MAF for three Chilean farmed Atlantic salmon populations with different origin. North America (NAM), Scottish (SCO), and Norwegian (NOR).

The 32K common markers are uniformly distributed along the 29 chromosomes, with an average SNP density per chromosome per Mb ranging from 8.39 to 20.55 with a mean of 14.10 ± 3.14 (Supplementary Table S1). All three populations showed a similar mean MAF of 0.26 ± 0.13, 0.29 ± 0.13, and 0.32 ± 0.12 for the NAM, SCO, and NOR, respectively. The mean MAF per chromosome ranged from 0.22 to 0.29 in the NAM population. For the populations with European origin, the MAF ranged from 0.24 to 0.31 and from 0.31 to 0.34 for SCO and NOR population, respectively. The proportion of loci with MAF higher than 0.20 ranged from 0.20 to 0.34 along the three populations. For those loci with MAF between 0.05 and 0.09, the proportion reached up to 0.13, 0.09, and 0.04 for the NAM, SCO, and NOR population, respectively (Figure 1).

### Population Structure

**FIGURE 2:**
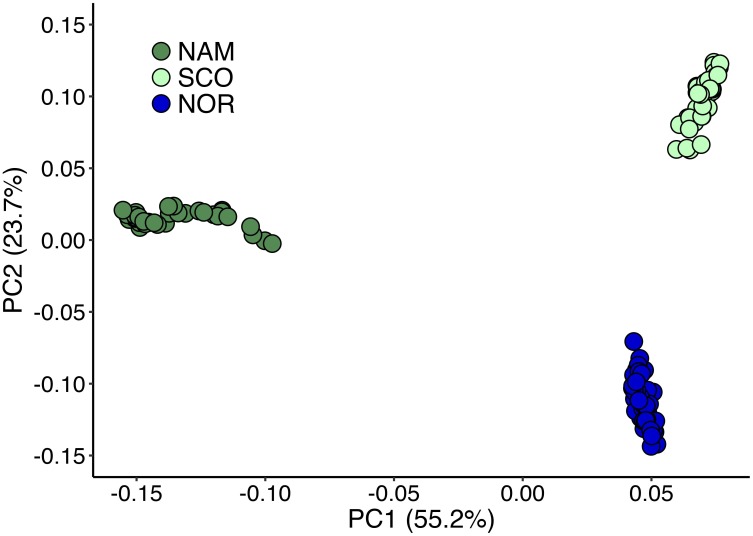
Genetic differentiation of Atlantic salmon populations revealed by principal component analysis. Principal component analysis for three Chilean Atlantic salmon breeding populations with different geographical origin. North America (NAM), Scottish (SCO), and Norwegian (NOR).

**FIGURE 3:**
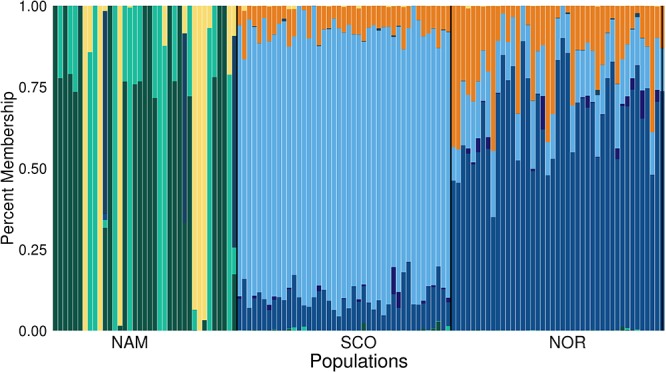
Population structure (*K* = 8) in three farmed populations of Atlantic salmon. Population structure in three farmed populations of Atlantic salmon with North America (NAM), Scottish (SCO), and Norwegian (NOR) origin. Each vertical line represents an individual, and each color a different theoretical ancestral population.

Principal components 1 and 2 together accounted for 78.9% of the total genetic variation (Figure 2). These components clearly revealed three different clusters, corresponding to the Atlantic salmon with North American (NAM), Scottish (SCO), and Norwegian (NOR) origin. The first principal component discriminates populations with North American and European origin and accounted for 55.2% of the total variation. The second principal component accounted for 23.7% of the total variance and divided the two European populations into two clusters, corresponding to Scottish and Norwegian populations, respectively. According to STRUCTURE analysis, using 31,978 common SNPs across three populations, we obtained the best *K* = 8 by computing the posterior probabilities of each K. NAM population presented the highest level of admixture, while SCO presented the lowest (Figure 3). STRUCTURE results assessing K values from 2 to 10 are presented in Supplementary Figure S1, while posterior probabilities are showed in Supplementary Table S2.

### Linkage Disequilibrium Decay

**FIGURE 4:**
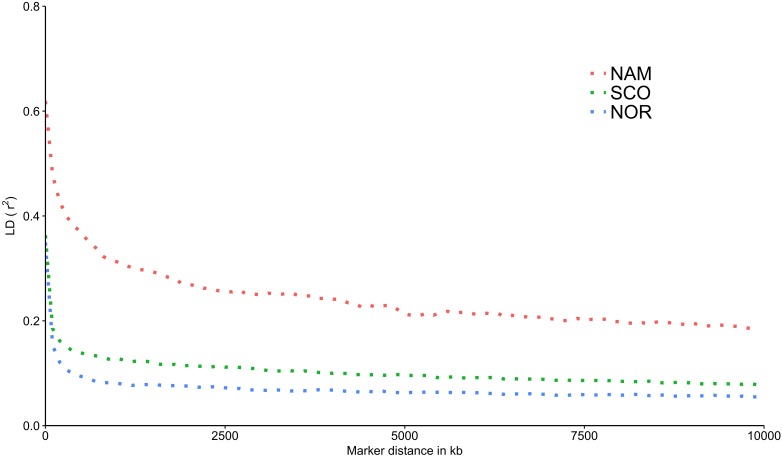
Decay of average linkage disequilibrium (*r*^2^) over distance in three farmed populations of Atlantic salmon. Average linkage disequilibrium between markers measured as *r*^2^ in three farmed populations of Atlantic salmon with North America (NAM), Scottish (SCO), and Norwegian (NOR) origin.

The LD decay was estimated for each population as a function of physical distance. SNP pairs were sorted in 100 kb-bins based on the distance between pairs. Average *r*^2^ values were estimated for each bin. As estimated in other domestic animals (Badke et al., 2012; Makina et al., 2015; Kijas et al., 2017; Barria et al., 2018a), genome-wide average LD declines with increasing physical distance between markers. Figure 4 shows an overview of the decay of *r*^2^ as a function of distance for each population. A slow decay was observed for the NAM population, while the decay was faster in both populations with European origin. The average distance at which the LD value reached 0.2, varied between populations. For the NAM population the distance reached ∼ 7,800 kb. For the SCO and NOR populations, the distance decreased drastically, corresponding to ∼ 64 and 50 kb, respectively. Average *r*^2^ for the first bin at distances of 0.5, 1.0, 5.0, and 10.0 Mb is shown in Table 3. Mean *r*^2^ within the first bin was larger for the NAM population (*r*^2^ = 0.62), followed by the SCO and NOR (*r*^2^ = 0.36 and 0.35, respectively). Average *r*^2^ for SNP pairs with a mean distance of 1.0 Mb was 0.31, 0.13, and 0.08 for the NAM, SCO, and NOR populations, respectively. These values decreased to 0.19, 0.08, and 0.06, when average distance between SNPs reached up to 10.0 Mb for NAM, SCO, and NOR, respectively.

**Table 3:** Mean linkage disequilibrium (*r*^2^) at different distances in three Chilean farmed populations of Atlantic salmon with North American (NAM), Scottish (SCO), and Norwegian (NOR) origin.

Population	0.1 Mb	0.5 Mb	1.0 Mb	5.0 Mb	10.0 Mb
NAM	0.62	0.37	0.31	0.21	0.19
SCO	0.36	0.14	0.13	0.10	0.08
NOR	0.35	0.09	0.08	0.06	0.06


### Effective Population Size

**FIGURE 5:**
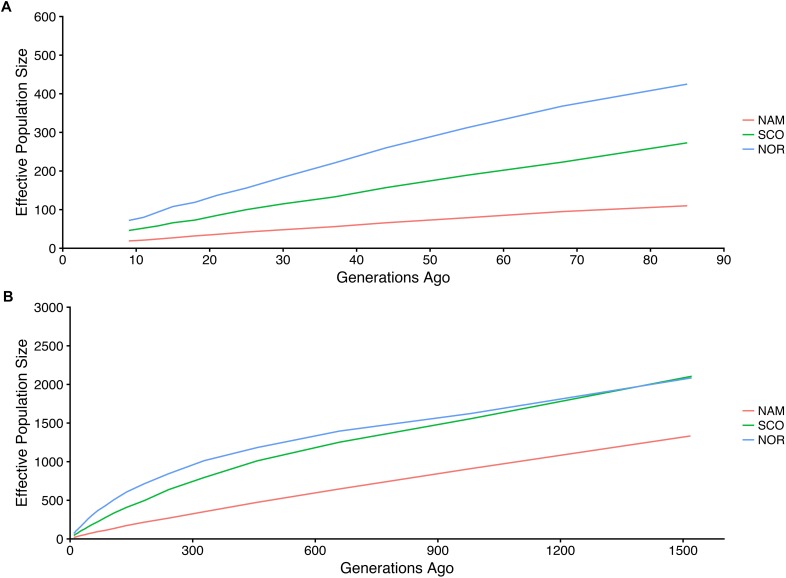
Estimated effective population size in three farmed populations of Atlantic salmon. Historic effective population size from 9 up to 85 **(A)** and 1516 **(B)** generations ago for three Atlantic salmon farmed populations with North America (NAM), Scottish (SCO), and Norwegian (NOR) origin.

Estimated effective population size differed among populations. Figure 5 shows the historical trends in *N*_e_ up to 85 (Figure 5A) and 1516 (Figure 5B) generations ago, respectively. Within this range of generations, Atlantic salmon with North American origin had the smallest *N*_e_, followed by SCO and NOR populations. These *N*_e_ values ranged from 15 to 574; 44 to 1,346; and from 72 to 1,325 for NAM, SCO, and NOR populations, respectively. Contemporary *N*_e_ estimations based on LD reached up to 7.5, 107, and 160 for NAM, SCO, and NOR populations, respectively.

## Discussion

The study of extent and decay of whole-genome LD can aid in the understanding of demographic processes experienced by populations. Processes such as founder effect, admixture and genetic drift in conjunction with recombination and mutations are key elements determining LD. Similarly, other factors that affect LD include inbreeding, admixture and selection (Gaut and Long, 2003); which has resulted in studies aimed at estimating LD variation between populations (Ai et al., 2013; Yang et al., 2014; Al-mamun et al., 2015; Mdladla et al., 2016).

This is the first study aimed at characterizing the decay and extent of LD in three different Atlantic salmon breeding populations established in Chile, representing the three main geographical origins of this cultured species now in Chile and worldwide. All three populations have been subjected to artificial selection for growth related traits. Individuals used in this study were selected having non-common ancestors for three generations back to avoid inflated LD estimations that are likely to occur due to high kinship relationships (Gutierrez et al., 2015).

The results presented here indicate the existence of differential average levels of LD across the genome between these Atlantic salmon populations. Large differences between North American and European populations were expected considering that they belong to two different lineages probably separated by more than 1,000,000 years (Rougemont and Bernatchez, 2018). The long-range LD found in the NAM population is likely a consequence of the admixture, as it has been suggested in Atlantic salmon (Ødegård et al., 2014; Rougemont and Bernatchez, 2018) and rainbow trout (Vallejo et al., 2018) populations. However, it could also reflect demographic process in the strain formation, as well as demographic events in its wild progenitors. It is well known that North American populations of Atlantic salmon have lower genetic diversity than European populations (Bourret et al., 2013; Makinen et al., 2014), as migrations probably favored only a few individuals colonizing North America, reducing effective population sizes and causing a major effect of genetic drift (Rougemont and Bernatchez, 2018) on patterns and degree of LD, especially in closely linked loci (Kruglyak, 1999). Additionally, artificial selection in the NAM population has been probably stronger than in SCO, presenting the former higher level of LD probably by the breeding process. On the other hand, the lowest level of LD found in NOR is consistent with its more diverse origin (Norris et al., 1999). The NOR population was established in early 1970s using fish from several rivers on the west coast in Norway, which probably favored greater genetic diversity than NAM and SCO.

Recent events of admixture decrease the short-range LD present in original populations (Ødegård et al., 2014) and haplotypes with high LD levels are shorter in highly admixed populations (Toosi et al., 2010). Admixture can also generate long-range LD, which could be captured by lower density SNP panels (Ødegård et al., 2014; Vallejo et al., 2018). Conversely, the highest overall levels of LD present in the SCO and NAM populations may be reflecting the unique origin of these populations without the recent introgression of different genetic material and a small effective population size, as it has been shown in Tasmanian Atlantic salmon (Kijas et al., 2017) and Pacific salmon (Barria et al., 2018a). On the other hand, we suggest that admixed origin of NOR population could cause an elevated extent of LD, but more than four decades of domestication and artificial selection have been enough to broke down the initial pattern and levels of LD in this population.

Our results suggest that the effect of these demographic features is more extreme in the NAM population, which has the highest level of LD of the three populations analyzed. In general terms, LD varied moderately between chromosomes in the NAM population, suggesting a variation in autosomal recombination rate which could be associated to genetic drift or artificial selection (Arias et al., 2009). Population analysis using STRUCTURE yielded the best *K* = 8, showing the highest level of admixture in NAM, which agrees with the hypothesis that the American population of Atlantic salmon was founded by multiple European sources (Rougemont and Bernatchez, 2018). Besides that, PCA shows clear genetic differentiation among the three populations, being PC1 that split NAM of European populations. This confirms the great divergence between European and North American Atlantic salmon, which agrees with STRUCTURE results, where it shows a clear differentiation between the NAM and both European populations.

The lower levels of SNP variability observed in the NAM population was expected, considering that North American salmon populations have lower genetic diversity than European populations (Bourret et al., 2013; Makinen et al., 2014). This also may be attributed to the ascertainment bias caused by prioritization of SNP markers segregating in NOR and SCO populations in the design of the SNP array used in the present study (Yáñez et al., 2016). A similar situation has been observed when evaluating performance of SNP panels, which have been designed to account for the variability in European populations of Atlantic salmon and in Tasmanian farmed Atlantic salmon populations with a North American origin (Dominik et al., 2010; Kijas et al., 2017). To reduce this bias in the genetic differentiation analysis, we used a common subset of SNPs of approximately 32 K. However, this does not ensure better estimates of genetic diversity, consequently these results should be interpreted with caution as suggested by Rougemont and Bernatchez (2018). Inaccurate estimates of genetic parameters due to ascertainment bias could be avoided by using a SNPs array developed with North American Atlantic samples specifically, that will provide more information about local variation.

It has been suggested that the minimum number of individuals needed for accurate LD estimations using *r*^2^ ranges between 55 and 75 individuals, increasing to more than 400 for |*D’*| (Khatkar et al., 2008; Bohmanova et al., 2010). However, an accurate estimation of LD measured as *r*^2^, has been obtained in Pacific salmon using 62 individuals (Barria et al., 2018a). Because of the relatively small sample size of each population (37, 43, and 43 for the NAM, SCO, and NOR populations, respectively), we measured LD decay as *r*^2^, instead of |*D’*|. Furthermore, estimates of *r*^2^ are less susceptible to overestimation and are more useful to predict the power of an association mapping (Ardlie et al., 2002; Bohmanova et al., 2010). Significant linear association has been assessed previously between chromosome length and LD (as *r*^2^) in Nellore cattle (Espigolan et al., 2013). We only found significance between these variables in the NOR population (*p* < 0.05). Like Bohmanova et al. (2010), we found no association in SCO and NAM populations, which could be due to lower marker density (data not shown).

The current results compared the LD decay between Chilean Atlantic salmon breeding populations originating from different geographic regions. The SNP panel used in the current study has one SNP every 14 kb (Yáñez et al., 2016). Based on a *r*^2^ threshold value of 0.2, as suggested by Meuwissen et al. (2001), reached at a minimum marker distance of 50 kb, this panel can be used to detect associations between markers and traits of interest and also capture high-resolution information for genome predictions.

## Conclusion

The current study reveals different LD decay between three Atlantic salmon farmed populations. The highest extent of LD was estimated for the NAM population, followed by the SCO and NOR populations. A lower level of LD in NOR was consistent with its population history. Specifically, this population comes from a farmed strain established with samples from several rivers in Norway. Therefore, subsequent genetic bottlenecks associated with strain formation have been less severe in comparison with the other two populations used in this study, that were established using fish from only one location. Also, the highest level of LD and lowest *N*_e_ that wa1s observed in NAM is consistent with the hypothesis that American salmon colonization from European fish favored only a few individuals. The high long range LD in NAM indicates the feasibility of achieving better prediction accuracies in this population with a smaller SNP data set than European populations.

## Data Availability

Raw genotype data for each population is available from the online digital repository Figshare, accession number doi: 10.6084/m9.figshare.7144631.

## Ethics Statement

The sampling protocol was previously approved by The Comité de Bioética Animal, Facultad de Ciencias Veterinarias y Pecuarias, Universidad de Chile (certificate N° 29–2014).

## Author Contributions

AB performed LD and *N*_e_ analyses, and wrote the initial version of the manuscript. ML performed populations structure analysis, first quality control of genomic data and contributed with discussion and writting. GY contributed with LD analysis and discussion. RC and JL contributed with analysis and discussion. JY and JL conceived and designed the study. JY supervised work of AB and contributed to the analysis, discussion, and writing. All authors have reviewed and approved the manuscript.

## Conflict of Interest Statement

The authors declare that the research was conducted in the absence of any commercial or financial relationships that could be construed as a potential conflict of interest.

## Footnotes

1 http://media.affymetrix.com/support/downloads/manuals/axiom_best_practice_supplement_user_guide.pdf
