# Supplementary figures and images for: Population Genomic Structure and Genome-Wide Linkage Disequilibrium in Farmed Atlantic Salmon (Salmo salar L.) Using Dense SNP Genotypes
Source: Front Genet. 2018 Dec 14;9:649. doi: 10.3389/fgene.2018.00649 (PMC6302115; doi:10.3389/fgene.2018.00649)

**Supplementary Figure 1.** Inferred population structure of samples using the program STRUCTURE

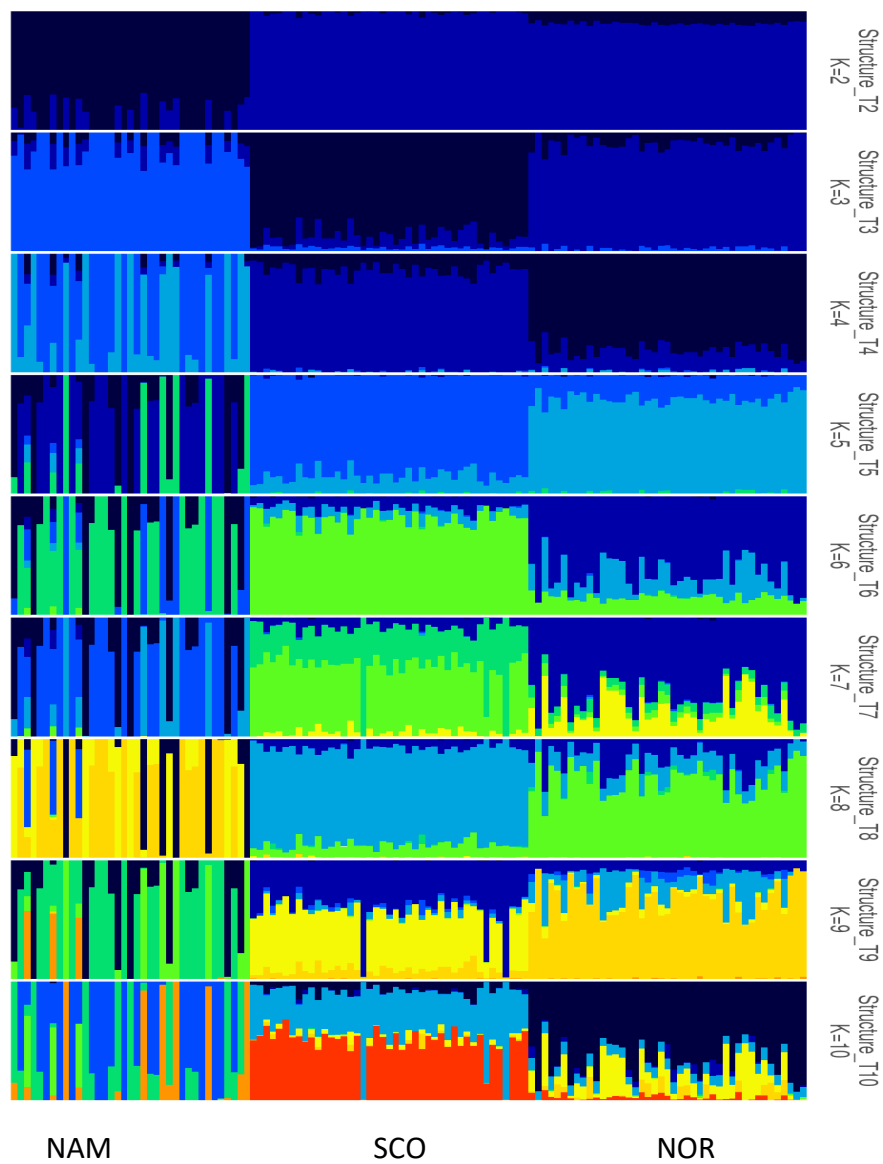

Supplement: Supplementary file 1 [file Image_1.pdf]
